# Short-Term Teriparatide for Bone Marrow Edema Secondary to Complex Regional Pain Syndrome: Case Reports on Efficacy After Two Years of Follow-Up

**DOI:** 10.7759/cureus.8119

**Published:** 2020-05-14

**Authors:** Felice Galluccio, Abdallah El Sayed Allam, Francesco Perdisa, Ke-Vin Chang

**Affiliations:** 1 Rheumatology - Experimental and Clinical Medicine, University of Florence, Florence, ITA; 2 Physical Medicine, Rheumatology and Rehabilitation, Tanta University Hospitals & Faculty of Medicine, Tanta University, Tanta, EGY; 3 Hip and Knee Replacement, IRCCS Istituto Ortopedico Rizzoli, Bologna, ITA; 4 Physical Medicine and Rehabilitation, National Taiwan University Hospital, Bei-Hu Branch, Taipei, TWN

**Keywords:** bone marrow edema, complex regional pain syndrome, knee injury, teriparatide

## Abstract

Bone marrow edema secondary to chronic pain syndrome after knee trauma is a disabling condition that presents with localized pain, allodynia, edema, decreased range of motion and osteopenia. The management includes a variety of medications and rehabilitation. The treatment of refractory diseases is challenging for most physicians. We present two cases of refractory bone edema secondary to complex regional pain syndrome that were successfully treated with a short-term regimen of teriparatide (TPT), a biosynthetic analogue (PTH 1-34) of the human parathormone. The rapid and sustained pain reduction with recovery of knee function for two years following administration of TPT demonstrated its potential for the treatment of bone edema due to complex regional pain syndrome.

## Introduction

Bone marrow edema (BME) is a generic term used to describe a common finding on magnetic resonance imaging (MRI) and can either present spontaneously (Spontaneous osteonecrosis, insufficiency fractures, etc.) or originate from various pathologies (secondary BME, sBME) [1]. Among the traumatic causes of sBME, complex regional pain syndrome (CRPS) is probably the most disabling, characterized by regional pain with allodynia or hyperalgesia, edema, vasomotor instability, movement disorders, joint stiffness, and regional osteopenia [2,3]. The diagnosis of CRPS is mainly clinical and is defined as continuous pain disproportional to the triggering trauma with the associated clinical signs (temperature asymmetry, skin color changes or trophic changes, edema and sweating, muscular weakness, tremor or dystonia) based on the Budapest criteria [4]. Despite different therapeutic approaches (corticosteroids, non-steroidal anti-inflammatory drugs (NSAIDs), calcitonin, opioids, analgesics, sympatholytic agents and sympathetic nerve and ganglion blockade, bisphosphonate and physical therapy), the natural history of post-traumatic bone contusions has been poorly investigated, especially at long-term [5-7]. The evolution of CRPS is influenced by several factors, but pain and functional loss may last for a long time and even for a whole life.

Teriparatide (TPT) is a recombinant synthetic version of the human parathyroid hormone. Officially indicated for the treatment of postmenopausal or glucocorticoids-induced osteoporosis, TPT may also increase bone mass in men with primary or hypogonadal osteoporosis, directly enhancing bone formation and quality, thus significantly reducing the risk of secondary fracture [8]. A first off-label use of TPT has been investigated for the treatment of aseptic bone non-unions with a good safety profile and promising preliminary result [9]. Moreover, it has been successfully applied in case of transient osteoporosis and osteonecrosis of the hip [10,11]. Among the causes of BME at the knee joint, those secondary to CRPS are less investigated, and limited treatment options are available up to date.

We present two cases of chronic sBME of the knee secondary to CRPS successfully treated with TPT with complete symptoms improvement and full recovery of the function.

## Case presentation

First case

**Figure 1:**
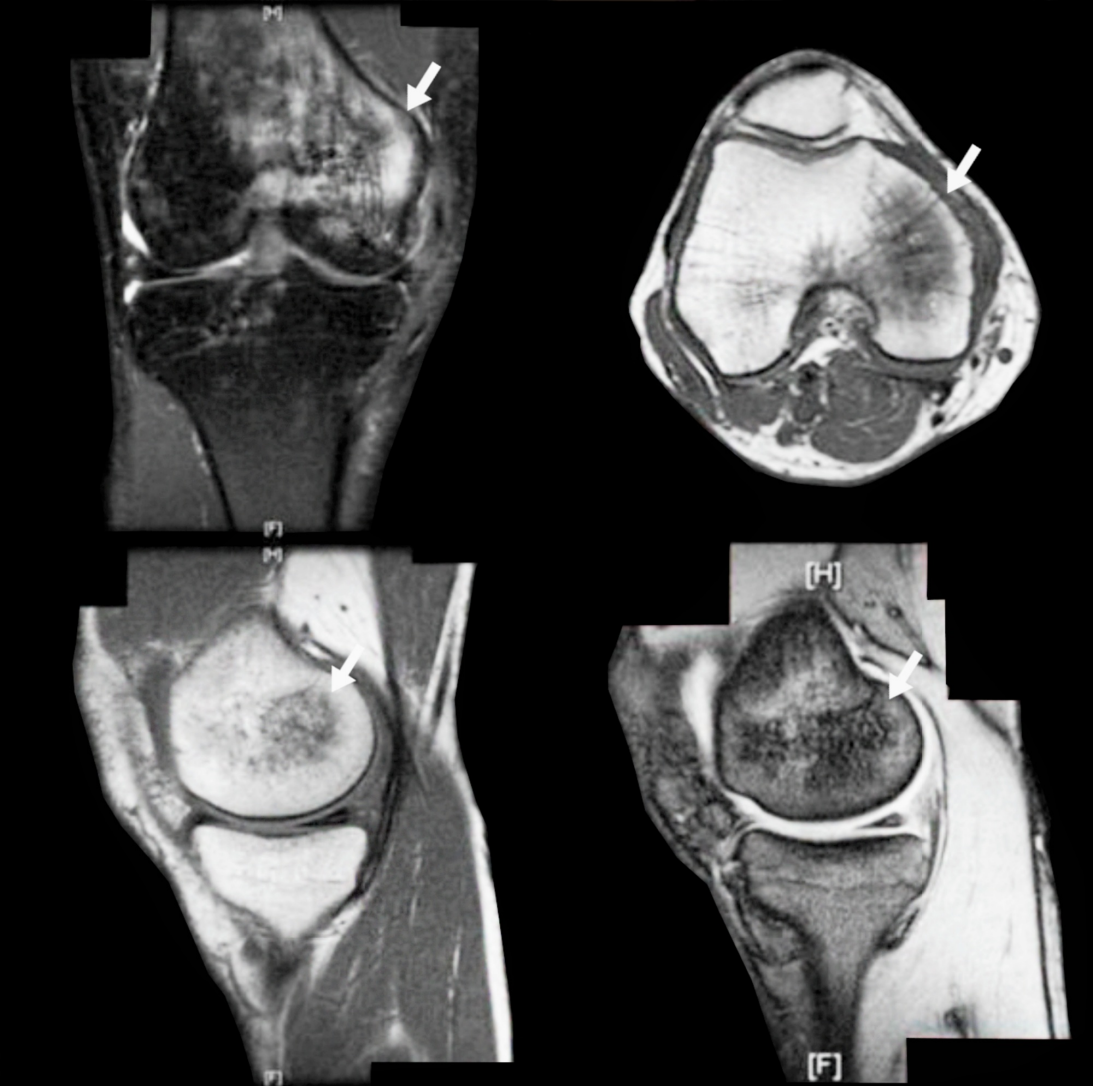
First case before teriparatide (TPT) treatment The arrows show area of bone edema.

A 44-year-old man attended our outpatients’ clinic for persistent right knee swelling after direct trauma occurred three months earlier. MRI, performed in the emergency room, showed extensive edema of the trabecular bone of the medial femoral condyle, without fractures lines. The patient was discharged with a plaster immobilization of the knee, protected weight-bearing and oral NSAIDs. During the immobilization period, the patient reported an increase in pain and paresthesia of the knee. At the first follow-up evaluation, the patient presented significant joint effusion and limited knee range of motion (ROM) without signs of knee instability. On the anteromedial aspect of the knee, the skin was atrophic and discolored, cold and with a peculiar area of allodynia. Under the impression of post-traumatic CRPS, MRI scan was performed to detect possible trabecular fractures, showing a large area of edema of the medial femoral condyle which was diagnosed as an algodystrophic modification (Figure 1).

**Figure 2:**
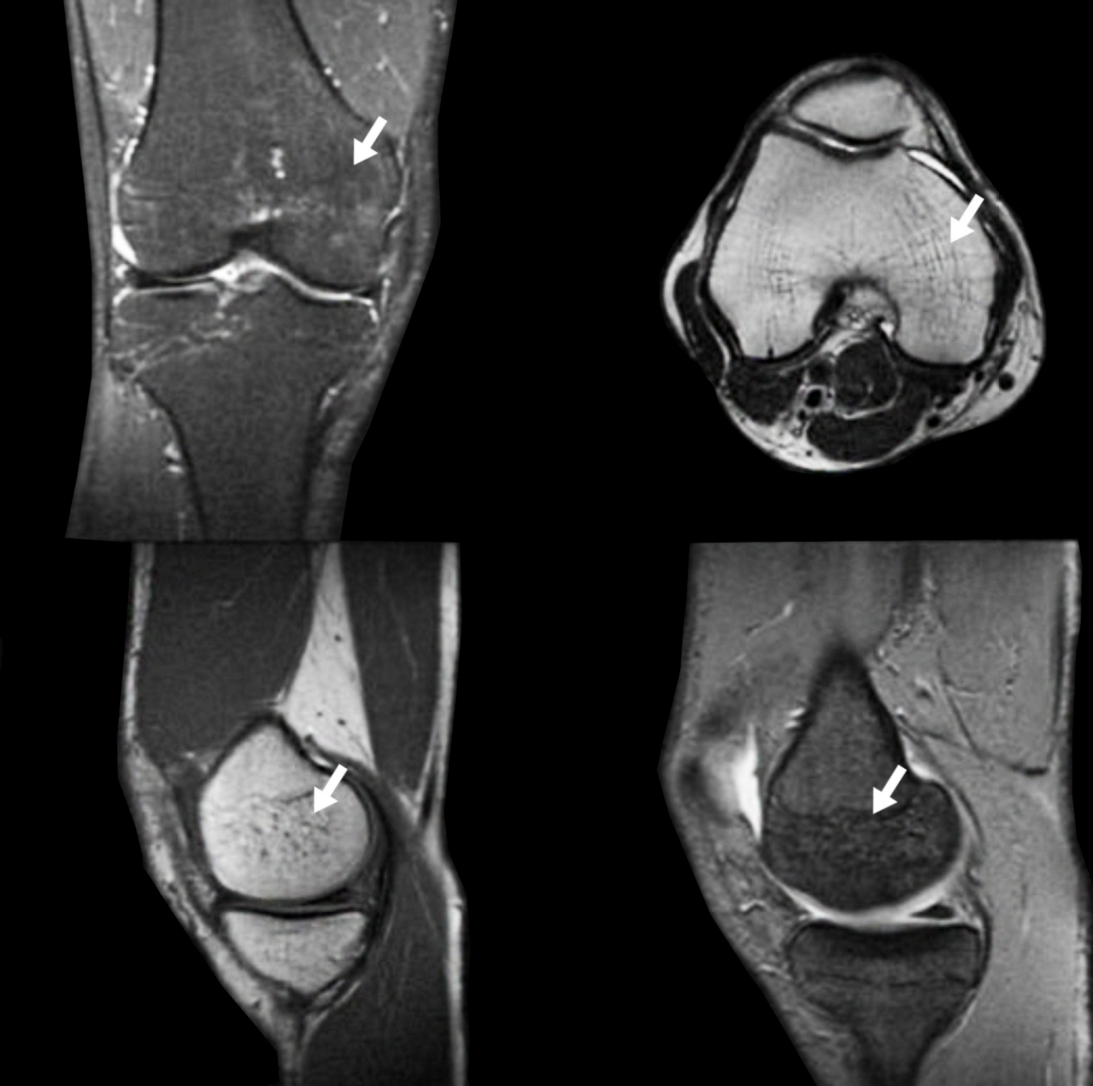
First case after teriparatide (TPT) treatment The arrows show resolution of the bone edema.

All diagnostic tests were performed to rule out other possible causes. A combined treatment with low dose oral prednisone, gabapentin 300 mg three times a day, daily calcium carbonate 1250 mg-cholecalciferol 400 UI and intramuscular clodronate 200 mg daily for 15 days was prescribed. Additionally, a rehabilitation program was initiated. The treatment was discontinued after four months due to the lack of any symptom’s improvement. Afterward, the patient underwent TPT (20 mcg day) and calcium carbonate/cholecalciferol (1250 mg/400 UI) supplementation. After one month, the patient reported a marked subjective improvement in terms of both pain and joint mobility. On clinical examination, no joint swelling was noticed, with almost complete recovery of range of motion and disappearance of the cold skin overlying the medial femoral condyle. The patient started physical training and the knee function was completely recovered after one month. After three months, MRI showed almost complete resolution of the joint effusion and bone edema (Figure 2).

TPT was then discontinued, but the rehabilitation program was maintained. At two years of follow-up, no recurrence of joint swelling and pain was reported, and the patient was back to regular sports and work activities.

Second case

**Figure 3:**
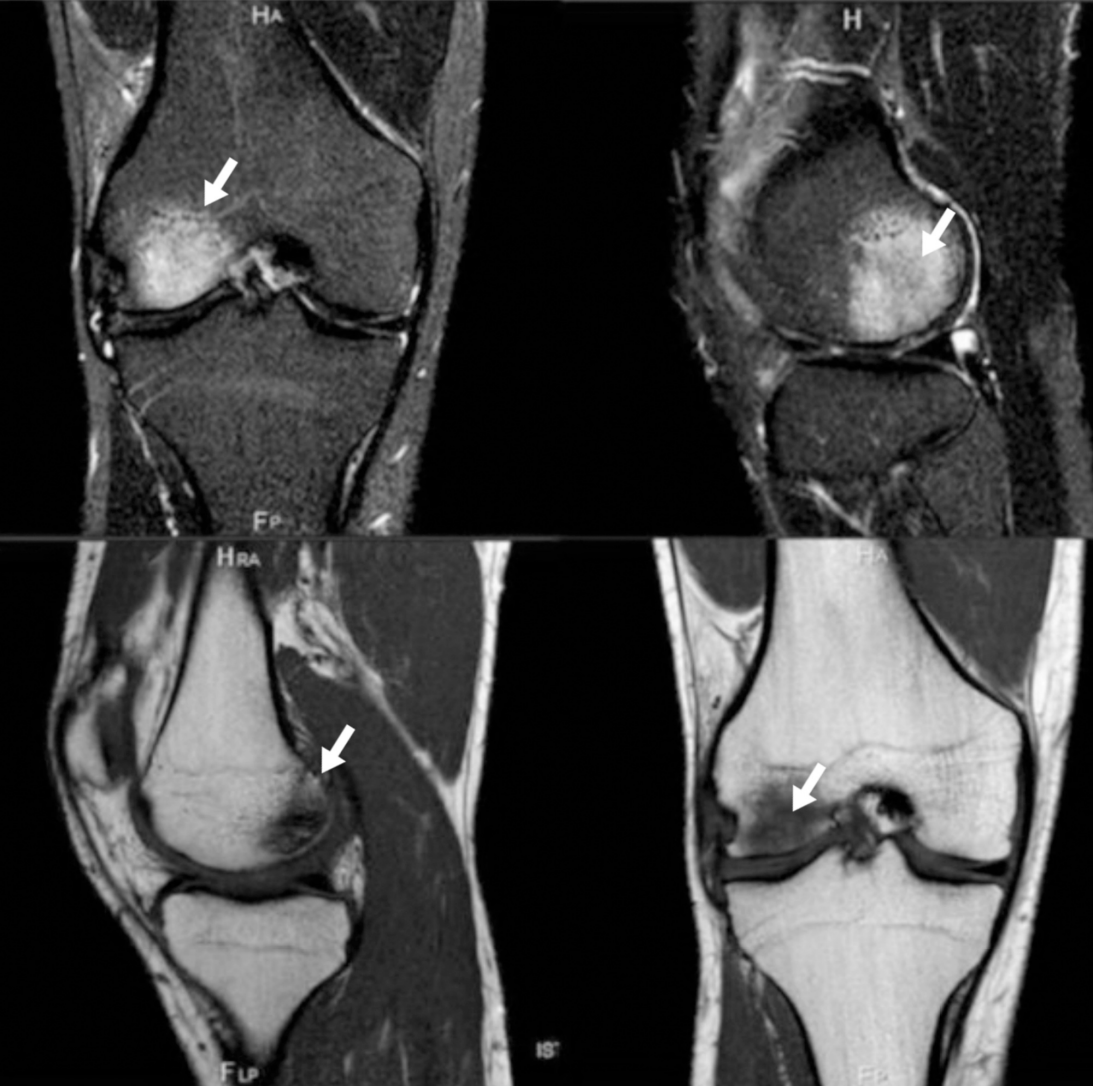
Second case before teriparatide (TPT) treatment The arrows show area of bone edema.

A 21-year-old football player was sent from the orthopedic clinic for persistent knee pain and swelling, started after trauma six months before. Neither prior history nor trauma or joint disease was detected. MRI showed joint effusion and osteochondral sufferance of the lateral femoral condyle. The patient was given etoricoxib (90 mg), glucosamine (1500 mg) and methylsulfonylmethane (5 gr/daily) at first, but a subjective worsening of symptoms was reported, together with onset of allodynia, hyperalgesia, and progressive loss of joint function. CRPS was suspected, and alendronate 70 mg/weekly and calcium carbonate - cholecalciferol supplement (1250 mg/400 UI) were prescribed but no improvement was noted. In fact, at the first evaluation, knee joint effusion with limited range of motion was present with difficulty to stand on the affected limb with area of allodynia and hypotrichosis on the knee. MRI showed diffuse progressive trabecular bone edema and bone resorption (Figure 3).

**Figure 4:**
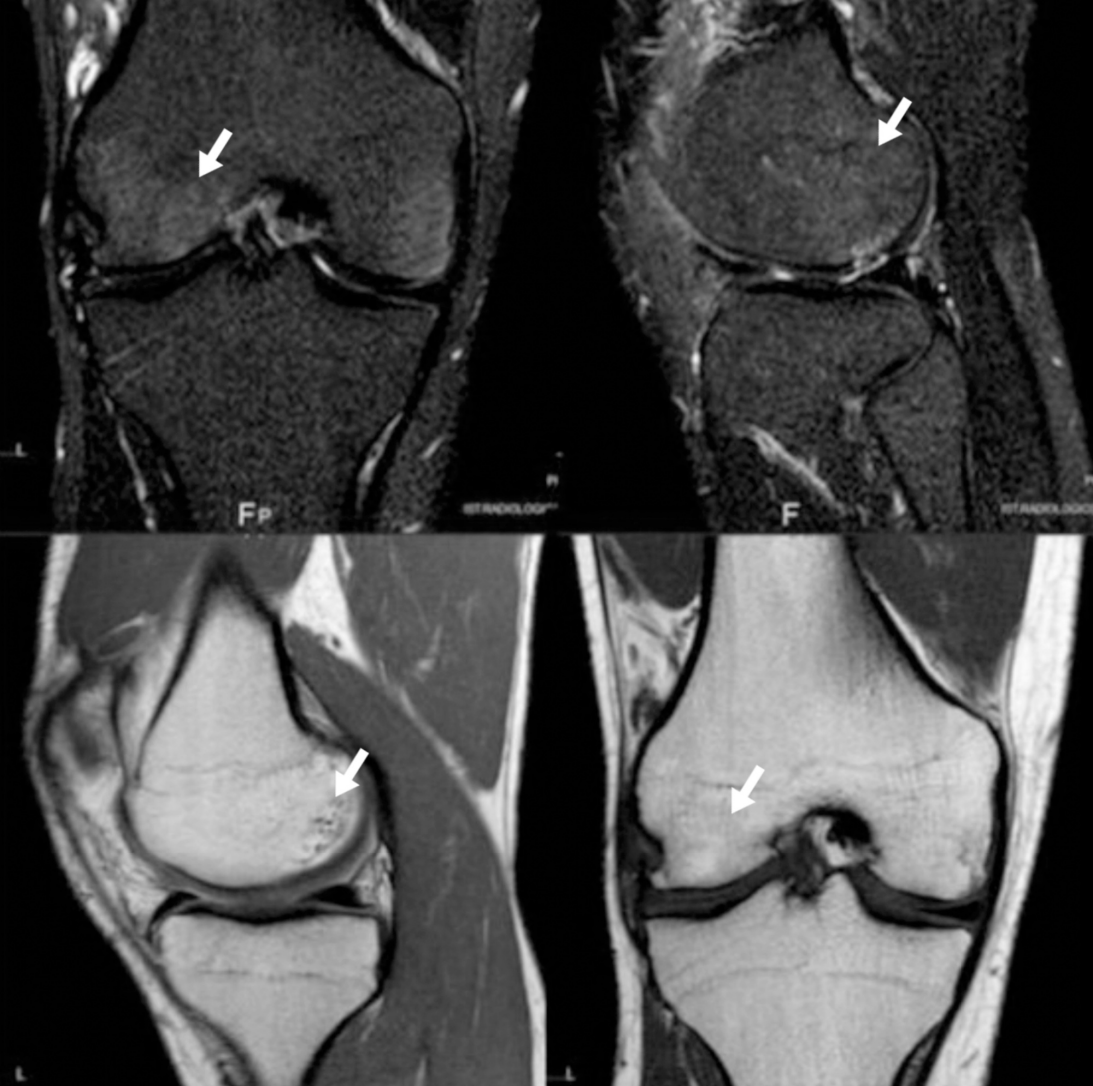
Second case after teriparatide (TPT) treatment The arrows show resolution of bone edema.

All laboratory tests (erythrocyte sedimentation rate, C-Reactive protein, parathormone, bone alkaline phosphatase, serum and urinary calcium/phosphate and 25(OH)D3) were normal. The patient later received daily TPT 20 mg daily for three months. A rehabilitation program was given to recover ROM and muscles tone of the affected knee. After the first month, only residual minimal effusion and muscle hypotrophy were presented and the patient was able to start strength training without suffering consequence. After three months, no joint effusion was detectable and complete recovery of knee function and muscles mass were evident. Control MRI showed trabecular bone edema nearly disappeared (Figure 4).

TPT was then discontinued. Neither side effects nor symptoms recurrence during and after discontinuation of treatment was experienced. At two years of follow-up, the patient can still conduct regular competitive sport activities.

## Discussion

Despite the increased scientific interest and numerous publications in the literature, the pathophysiology of sBME and CRPS is still not clarified. In fact, CRPS includes a variety of pathological processes ranging from central and peripheral sensitization, sympathetic dysfunction, abnormal inflammation with overproduction of proinflammatory cytokines and neuropeptides, enhanced bone resorption and turnover to aberrant healing. The clinical picture and the symptoms are often highly variable across cases, especially for those after trauma the treatment strategy is still challenging for most physicians. Thus, the choice of the most suitable therapy depends individual experience and expertise. In our report, the patients were initially treated with NSAIDS, bisphosphonate (clodronate and alendronate) and joint immobilization but the treatment failure led us to try TPT for a short-term period (three months). The rationale of TPT use is based on the regulatory function of parathormone. In fact, TPT is a recombinant biosynthetic version of the N-terminal of human PTH, is an anabolic agent directly stimulating bone formation and improving bone mass and quality [12]. It has been demonstrated that the maximum anabolic effect is obtained in the first period of treatment while bone remodeling ensues [13]. Other factors, like the up-regulation of growth factors including basic-fibroblast growth factor-2 (bFGF-2) and insulin-like growth factor-1 (IGF-1), receptor activator of nuclear factor-kappa B ligand (RANKL), the influence on the Wnt/β-catenin signaling pathway and finally the transcriptionally suppression of the sclerostin gene may play an important role on the anabolic and regulating effect of TPT on bone [14-16]. As previously reported, TPT has an adrenal effect leading to an increase of plasma and urinary cortisol during the treatment period [17]. The importance of this effect is still unclear but it may have a regulatory effect on the inflammation cascade. Many studies have shown that TPT accelerated fracture healing and has also a potential effect in increasing bone volume within joints and inhibiting articular cartilage degeneration [18-20].

TPT may play a key role in patients with BME secondary to CRPS refractory to standard treatments regarding its sustained effect in pain reduction and knee functional improvement. The rapid response to TPT and the possibility to discontinue it after only three months may be due to not only in its anabolic properties, but also to the ability to regulate the “bone system” including cell signaling and network.

One limitation of this case reports is that we cannot exclude the influence of previous therapy or a spontaneous resolution of the disease. However, the immediate improvement after starting TPT compared to the ineffectiveness of the previous drugs could be considered a direct effect. In refractory cases of BME secondary to CRPS, as well as seen for hip osteonecrosis, TPT might be considered as an alternative treatment, although further confirmation is needed [11].

## Conclusions

The rapid and sustained pain reduction with recovery of knee function for two years following administration of TPT demonstrated its potential in treatments of bone edema due to complex regional pain syndrome.
